# Significant decrease in plasmad‐dimer levels and mean platelet volume after a 3‐month treatment with rosuvastatin in patients with venous thromboembolism

**DOI:** 10.1002/clc.23833

**Published:** 2022-04-28

**Authors:** Toktam Alirezaei, Haniyeh Sattari, Rana Irilouzadian

**Affiliations:** ^1^ Clinical Research Development Unit of Shohada‐e Tajrish Hospital Shahid Beheshti University of Medical Sciences Tehran Iran; ^2^ Department of Cardiology, School of Medicine Shahid Beheshti University of Medical Sciences Tehran Iran

**Keywords:** inflammation, rosuvastatin, statin, venous thromboembolism

## Abstract

**Background:**

Inflammation has been considered as a possible mechanism for the initiation and recurrence of venous thromboembolism (VTE). Statins have anti‐inflammatory and potential immune‐modulatory effects, but their effect on plasmad‐dimer levels is controversial.

**Hypothesis:**

In this study, we aimed to evaluate the impact of rosuvastatin on D‐dimer and other inflammatory serum markers in VTE patients.

**Methods:**

We conducted a prospective, randomized study on 228 patients with VTE. Control group received conventional treatment (warfarin or rivaroxaban), whereas rosuvastatin‐intervention group received rosuvastatin 10 mg daily, in addition to their conventional treatment for 3 months. Serum markers were extracted from both groups at the baseline and 3 months after the beginning of treatment.

**Results:**

After 3 months, in patients of the intervention group, there was a statistically significant decrease in levels ofd‐dimer and mean platelet volume (MPV) but no significant change in neutrophil‐to‐lymphocyte ratio and platelet‐to‐lymphocyte ratio.

**Conclusions:**

Our results showed that a 3‐month treatment with 10 mg rosuvastatin daily can significantly decrease the plasma levels ofd‐dimer and MPV, which would support a potential role of statins to reduce activated systemic inflammation among VTE patients. Such effects can be used to reduce the rate of recurrent VTE in these patients.

## 1 INTRODUCTION

Venous thromboembolism (VTE), after myocardial infarction and ischemic stroke, is the third common vascular disease that has difficulties in management and high risk of recurrence. VTE term comprises of pulmonary embolism (PE) and deep vein thrombosis (DVT), along with an increased rate of mortality and morbidity.^1^ The prevalence of VTE has raised recently and it is estimated to affect 1–2 per 1000 people annually with ~2.5% mortality.^2^ VTE is a multifactorial and life‐threatening event accompanied by activation of coagulation and fibrinolysis. Development of VTE is suggested to arise from coagulation as the main contributor and inflammation, which has a pivotal role in development of VTE.^3^, ^4^ Accumulation of reactive oxygen species (ROS) leads to the deregulation of immune‐related biomarkers such as neutrophils, which negatively affect neutrophil‐to‐lymphocyte ratio (NLR). Increased inflammation, NLR and platelet–lymphocyte ratio (PLR) are considered to be associated with VTE progression. Higher risk of VTE recurrence and mortality has been found to be relevant to elevated NLR and PLR.^5^, ^6^

Elevated mean platelet volume (MPV) is a sign of increased inflammation, which increases the risk of cardiovascular disorders. Increased MPV levels have been reported in patients with chronic diseases with high mortality and morbidity.^7^ Higher MPV is considered as a risk factor for hypercoagulability and a prognostic marker for VTE risk.^8^, ^9^ d‐dimer, one of the fibrin‐based protein fragments, can be significantly found in plasma when blood clot is forming and its elevated levels is an index for the diagnosis of VTE.^10^, ^11^ Further, after discontinuation of anticoagulants in VTE patients, increased d‐dimer levels may propose hypercoagulability.^12^

Anticoagulants remain the conventional first‐line strategies for the prevention and treatment of VTE, even though they often have their own complications.^13^, ^14^ Statins (3‐Hydroxy‐3‐methylglutaryl coenzyme A reductase inhibitors) are extensively applied against the dyslipidemia to prevent possible stroke or cardiovascular events. It has been shown that statins might have antithrombotic effect without bleeding complications. As a result, they can be an alternative for VTE prophylaxis.^15^

The anti‐inflammatory properties of statins have not been fully understood yet; however, some investigations reported that they are able to mitigate the inflammatory mediators such as interleukin (IL)−1β in neurodegenerative disorders.^16^ By reducing neuro‐inflammatory responses, atorvastatin was able to inhibit amyloid‐β aggregation in rats with Alzheimer's disease. Simvastatin was reported to reduce inflammatory mediators such as IL‐1β, IL‐6, and tumor necrosis factor‐α.^17^ Similarly, rosuvastatin with different dosages has been reported to attenuate nicotinamide adenine dinucleotide phosphate oxidase‐dependent superoxide production in insulin‐resistant animals with diabetes type II.^18^ Rosuvastatin decreased the risk of VTE in patients with or without VTE risk factors.^19^ Further, in patients with previous VTE, daily consumption of 20 mg rosuvastatin was able to stop thrombin generation and decreased‐dimer and coagulation factors such as VII, VIII, and XI.^20^

Based on the beneficial effect of statins and their alternative roles as anti‐inflammatory and antiplatelet, herein we aimed to investigate the effect of 3‐month rosuvastatin consumption on blood‐based biomarkers in patients with VTE in an educational hospital in Tehran, Iran.

## 2 MATERIAL AND METHODS

### 2.1 Participants

Current clinical trial was carried out on 228 unprovoked VTE patients, aged 20–75 years, who are referred to the hospital and had no definite indication for initiating statin therapy. The exclusion criteria were as follows:

- Diagnosis of provoked VTE.
- Any history of inflammatory disorders and acute infections.
- Previous blood‐related disorders affecting neutrophils, lymphocytes, and platelets.
- Any previous anticoagulants consumption, history of antiplatelet, and statin consumption over a period of 6 months.
- History of heart failure, hepatic failure, coagulopathy, hyperlipidemia or previous VTE, cancer and hemodialysis.
- Indication for initiating statin therapy based on 2018 American Heart Association/American College of Cardiology guideline on the management of blood cholesterol.^21^

All participants provided written informed consent. The protocol and consent forms were approved by the institutional review board at Shahid Beheshti University of Medical Science. Trial protocol has been approved under registration reference IRCT20210416050990N1. Eligible participants were randomized into two groups in a 1:1 ratio: control group and intervention group. A centralized computer based system was used for randomization. They were followed up for 3 months. Follow‐up after hospital discharge was made by telephone calls or visit in clinics.

### 2.2 Data collection

Demographic and clinical data including personal interviews, questionnaires, and medical records were all collected. At the baseline and 3 months after intervention,d‐dimer, MPV, neutrophil, lymphocyte, and platelet counts were measured using enzyme‐linked immunosorbent assay, and NLR and PLR were calculated for all the participants. In addition, PE Severity Index (PESI) and Wells scores were calculated.

PESI score is a clinical prognostic score that classifies PE patients into five classes with increasing risk of mortality and is consisted of different variables: age, sex, history of cancer, heart failure, chronic lung disease, altered mental status, respiratory rate, heart rate, systolic blood pressure, arterial oxygen saturation, and temperature. We calculated PESI score as follows:

Patient's age in years if more than 80 years old summed with points received from these 10 criteria:

Male gender (10 points), history of heart failure (10 points), history of chronic lung disease (10 points), history of malignancy (30 points), altered mental status (60 points), systolic blood pressure < 100 mmHg (30 points), heart rate > 109 beats per minute (20 points), respiratory rate > 29 breaths per minute (20 points), body temperature < 36°C (20 points), and arterial oxygen saturation < 90% (20 points). Then we categorized patients as follows: PESI I (<65 points), PESI II/III (66–105 points), and PESI IV/V (>106 points).^22^, ^23^ Wells score is a clinically validated scoring system to predict the risk of PE occurrence in patients. Score ≤ 4 are categorized as PE unlikely and Score > 4 as PE likely. We measured Wells score as follows: hemoptysis (1 points), cancer (1 points), previous PE or DVT (1.5 points), heart rate > 100 beats per minute (1.5 points), recent surgery or immobilization (1.5 points), clinical signs of DVT (3 points), and alternative diagnosis less likely than PE (3 points).

Based on sex, age, type of VTE, PESI and Wells scores, and inclusion criteria, patients were divided into two groups, including control group, consisting of only anticoagulant‐receiver group (warfarin or rivaroxaban) and rosuvastatin‐receiver group (warfarin or rivaroxaban + 10 mg rosuvastatin). Blood samples were collected from both groups at the baseline and at 3 months after the beginning of treatment, and the mentioned biomarkers were extracted. The primary endpoint of this study was to evaluate the change in the mean plasma concentration ofd‐dimer following 3‐month treatment with 10 mg rosuvastatin. The secondary endpoints were to evaluate the variation in mean plasma levels of MPV, NLR, and PLR following 3‐month treatment with 10 mg rosuvastatin.

### 2.3 Data analysis

Data were analyzed using SPSS v21 software (SPSS, Inc.) and normality of data were assessed by Kolmogorov–Smirnov test. For describing qualitative variables, number and percent were used, and for describing quantitative variables, mean and SD were used. The relationship between variables was measured using Pearson coefficient correlation. In addition, *χ* ^2^ test was performed to compare the frequency distribution of nominal variables in the study groups and independent *t* test was used to compare the mean of the quantitative variable in the study groups. Then, *p* < .05 was considered significant in all tests.

## 3 RESULTS

Demographic data indicated that 50% of participants (*n* = 114) were women. Approximately 61% of all participants were smokers. In addition, 29.4% of patients had diabetes mellitus and 14% had hypertension (HTN). Table 1 showed the demographic characteristics of patients in both control and intervention groups. As the results showed, the most prevalent disease in these patients are diabetes (%55) and HTN (%50; Table 1).

**Table 1:** Baseline characteristics of control and intervention groups

	Total (*n* = 228)	Control (*n* = 114)	Intervention (*n* = 114)	*p*
Age, years, mean ± SD	49.1 ± 12.6	48.7 ± 12.6	49.6 ± 12.7	.59
Sex				
Male, *n* (%)	114 (50%)	59 (51.8%)	55 (48.2%)	.59
Female, *n* (%)	114 (50%)	55 (48.2%)	59 (51.8%)	.59
BMI, kg/m^2^, mean ± SD	29.4 + 5.6	29.4 ± 5.9	29.4 ± 4.9	.99
DM, *n* (%)	67 (29.4)	30 (44.8%)	37 (55.2%)	.12
HTN, n (%)	32 (14%)	16 (50%)	16 (50%)	1
DM and HTN, *n* (%)	24 (10%)	13 (54%)	11 (46%)	.8
Smoking, *n* (%)	140 (61%)	72 (51.4%)	68 (48.6%)	.58
PESI score	88.4 ± 18.4	84.2 ± 18.6	92.4 ± 17.6	–
Wells score	4.5 ± 0.9	4.4 ± 0.9	4.1 ± 1.1	.50
DVT				.64
Upper limb	3	2 (66.7%)	1 (33.3%)	
Proximal lower limb	44	24 (54.5%)	20 (45.5%)	
Distal lower limb	72	37 (51.4%)	35 (48.6%)	
Pulmonary thromboembolism				.23
Massive	12	6 (50%)	6 (50%)	
Submassive	38	14 (36.8%)	24 (63.2%)	
Low risk	88	47 (53.4)	41 (46.6%)	
Anticoagulant treatment				.21
Warfarin, *n* (%)	50 (21.9%)	21 (42%)	29 (58%)	
Rivaroxaban, *n* (%)	178 (78.1%)	93 (52.2%)	85 (47.8%)	

*Note*: Results were reported as mean ± SD. Independent *t* test was used to compare data.

Abbreviations: BMI, body mass index; DM, diabetes mellitus; DVT, deep vein thrombosis; HTN, hypertension; PESI, pulmonary embolism severity index.

According to our results, there was no significant differences (*p* > .05) in the frequency of gender, smoking, underlying diseases, mean age and body mass index (BMI) between the control and rosuvastatin groups. Based on PESI score calculation, the control (mean PESI score = 84.19 ± 18.6) and intervention groups (mean PESI score = 92.42 ± 17.57) were both categorized in the same category (PESI II/III = 66–105 points). Both control (Wells score = 4.39 ± 0.90) and intervention groups (Wells score = 4.050 ± 1.07) were considered as likely to develop PE, based on Wells criteria without any statistically significant difference (*p* > .05).

The differences in main variables between rosuvastatin and control groups have been represented in Table 2. Based on our results, after 3‐month intervention, the mean plasmad‐dimer levels in rosuvastatin group was significantly lower than control group (*p* < .001). In addition, 3‐month rosuvastatin treatment showed a significant reduction in MPV in intervention group (*p* < .001). However, other variables including NLR and PLR showed no significant differences between the control and rosuvastatin groups (*p* > .05) (Table 2).

**Table 2:** Laboratory test results of intervention and control groups

	Baseline			3 Months later		
	Control	Intervention	*p*	Control	Intervention	*p*
d‐dimer (µg/L)	695.7 ± 149.8	729.4 ± 149.0	0.08	447.5 ± 114.4	376.4 ± 107.4	0.001^a^
MPV (fl)	9.6 ± 0.7	9.6 ± 0.7	0.71	9.3 ± 0.9	8.6 ± 0.7	0.001^a^
NLR	2.3 ± 1.1	2.6 ± 1.6	0.17	2.5 ± 1.3	2.2 ± 1.0	0.06
PLR	139.1 ± 71.9	128.3 ± 62.8	0.22	134.9 ± 68.3	127.0 ± 56.0	0.33

*Note*: Results were reported as mean ± SD. Independent *t* test was used to compare data.

Abbreviations: MPV, mean platelet volume; NLR, neutrophil to lymphocyte ratio; PLR, platelet to lymphocyte ratio.

^a^ Significance level < .05 is considerable.

## 4 DISCUSSION

The risk of VTE recurrence increases after the first event. Many studies have indicated that the rate of VTE recurrence would be increased within the first 3 months, despite receiving anticoagulant treatment.^24^ Prandoni et al.,^25^ after a long‐term follow‐up of 1626 patients, reported 22% of VTE recurrence.

Numerous studies have demonstrated that statins can reduce the VTE incidence in high risk patients, as well as recurrent VTE and mortality in patients with a previous VTE.^26^, ^27^ Glynn et al.,^28^ in their meta‐analysis, mentioned that 20 mg daily rosuvastatin, significantly reduced the rate of VTE recurrence, indicating antiplatelet properties of rosuvastatin. In a study by Siniscalchi et al.,^29^ statin consumption with anticoagulants for VTE was associated with a 20% decrease in mortality and there was no difference between different drugs of statin class.

High levels ofd‐dimer have been reported in patients with residual vein occlusion.^30^, ^31^ In patients with VTE, higher amounts of d‐dimer will increase the risk of recurrent VTE by 30% for the first 6 months.^32^ Consistent with our results, Biedermann et al.^33^ in 2018 demonstrated that 20 mg/day rosuvastatin was able to significantly reduce the risk of thrombosis andd‐dimer levels in patients with previous VTE.^33^ Although our results showed a significant reduction ind‐dimer levels in rosuvastatin group, Sahebkar et al.^34^ demonstrated that hydrophilic features of statins such as pravastatin and rosuvastatin could not attenuate bloodd‐dimer significantly, whereas lipophilic‐based statins could reduced‐dimer levels after 3 months. A study by Wang et al.^35^ in 2021 showed that a 3‐month regimen of anticoagulation and atorvastatin was not able to reduced‐dimer levels, in comparison with the control group. However, due to low recruitment rate, they terminated the study earlier than their plan.^35^ Contrarily, our study enrolled 228 patients and demonstrated that some blood biomarkers such as mean plasmad‐dimer and MPV were significantly reduced in the intervention group that consumed warfarin or rivaroxaban, and 10 mg rosuvastatin for 3 months.

In a meta‐analysis by Ji et al.^36^ in 2019, MPV was reduced significantly after statin therapy which exhibited their antiplatelet properties. Statins are able to keep the proinflammatory cytokines in a normal range and are associated with reduced activity of platelets resulting in significant changes in MPV.^7^ In another study, antiplatelet properties of rosuvastatin significantly attenuated the MPV; also, the reduction of MPV was not associated with the changes in serum lipids, which suggested antiplatelet effect of statins, irrespective of their lipid‐lowering effect.^37^

The statin mediatory effects on lymphocyte levels have been reported previously. Simvastatin at a dose of 20 mg/day has been revealed to reduce plasma lymphocytes after 8 weeks.^38^ Likewise, Tunçez^39^ reported that 80 mg atorvastatin or 40 mg rosuvastatin could attenuate the NLR levels after 4 weeks; however, this reduction was not statistically significant.^39^ In line with our results, Gungoren et al.^40^ reported that a 24‐week statin therapy in 261 patients did not change the NLR significantly.^40^ In parallel with these studies, our data revealed that 3‐month consumption of 10 mg rosuvastatin did not change the NLR and PLR significantly.

There are some points in our randomized clinical trial that needs consideration. The patients and the physicians were not blinded to the treatment but the laboratory technicians were blinded to the samples. As a result, it is unlikely that it would affect the outcomes. Also, recurrent VTE was diagnosed by a blinded physician, which reduced the risk of detection bias. In another aspect, there was no significant differences in the frequency of gender, smoking, underlying diseases, mean age, BMI, PESI, and Wells score between control and intervention groups; however, there is still a trivial possibility of residual confounding. Both our control and intervention group consumed anticoagulation, which might influence the effect of rosuvastatin on recurrent VTE. In the end, our results were associated with rosuvastatin and attribution to other statins warrants further studies.

## 5 CONCLUSIONS

Asd‐dimer and MPV are elevated during inflammation and hypercoagulable states, decreasing these factors would be a proper goal to decrease VTE recurrence. Conventional treatment of VTE (warfarin or rivaroxaban) combined with 10 mg rosuvastatin daily for 3 months decreasedd‐dimer and MPV levels significantly, in comparison with the patients receiving only anticoagulant therapy. Our data support the use of rosuvastatin in addition to conventional anticoagulant therapy in treatment of VTE. Further studies with bigger study groups are warranted to validate our results and propose underlying mechanisms of this effect.

### 5.1 Clinical trial registration information

The protocol and consent forms were approved by the institutional review board at Shahid Beheshti University of Medical Science. Trial protocol has been approved in Iranian Registry of Clinical Trials (IRCT), which is a primary registry in the World Health Organization registry network set up, under registration reference IRCT20210416050990N1 (https://www.irct.ir/trial/55647).

### 5.2 Clinical perspective

- As prolonged elevation ofd‐dimer levels after VTE propose ongoing hypercoagulability and VTE recurrence, medications that decreased‐dimer levels can decline the rate of recurrence.
- A course of 3‐month rosuvastatin can decreased‐dimer and MPV levels significantly among VTE patients, reducing the risk of VTE recurrence.
- The anti‐inflammatory properties of statins without bleeding complications suggests a complementary treatment for VTE recurrence.

## CONFLICTS OF INTEREST

The authors declare no conflicts of interest.

## Supporting information

Supporting information.

## Data Availability

The data that support the findings of this study are available on a reasonable request from the corresponding author.
