# Supplementary material for: Significant decrease in plasmad‐dimer levels and mean platelet volume after a 3‐month treatment with rosuvastatin in patients with venous thromboembolism
Source: Clin Cardiol. 2022 Apr 28;45(7):717–22. doi: 10.1002/clc.23833 (PMC9286331; doi:10.1002/clc.23833)
Supplement: Supplementary file 1 — Supporting information. [file CLC-45-717-s001.doc]

**CONSORT 2010 Flow Diagram**

**Allocation**

**Analysis**

**Follow-Up**

**Enrollment**

Assessed for eligibility (n=288)

Excluded (n=36)

♦  Not meeting inclusion criteria (n=31)

♦  Declined to participate (n=0)

♦  Died due to massive PTE (n= 5)

Analysed (n=114)
♦ Excluded from analysis (n=0)

Lost to follow-up (retired consent) (n=4)

Discontinued intervention (Did not consume drugs) (n=2)

Allocated to anticoagulant and rosuvastatin (n=126)

♦ Received allocated intervention (n=120)

♦ Did not receive allocated intervention (later refused to consume the drug) (n=6)

Lost to follow-up (retired consent) (n=6)

Discontinued intervention (Unknown reasons) (n=2)

Did not consent for blood sample collection (n=4)

Allocated to anticoagulant (n=126)

♦ Received allocated intervention (n=126)

♦ Did not receive allocated intervention (give reasons) (n=0 )

Analysed (n=114)
♦ Excluded from analysis (n=0)

Randomized (n=252)
